# Self‐help group participation, avoidance of nonalcoholic beer, and nonsmoking independently predict better drinking outcomes in Japanese alcohol‐dependent men

**DOI:** 10.1111/acer.70227

**Published:** 2026-02-01

**Authors:** Akira Yokoyama, Mitsuru Kimura, Atsushi Yoshimura, Junichi Yoneda, Hitoshi Maesato, Yasunobu Komoto, Hideki Nakayama, Hiroshi Sakuma, Yosuke Yumoto, Tsuyoshi Takimura, Tomomi Toyama, Takeshi Mizukami, Tetsuji Yokoyama, Susumu Higuchi, Sachio Matsushita

**Affiliations:** ^1^ National Hospital Organization Kurihama Medical and Addiction Center Yokosuka Japan; ^2^ Division of Psychiatry Tohoku Medical and Pharmaceutical University Sendai Japan; ^3^ Department of Psychiatry National Hospital Organization Ryukyu Hospital Kunigami Japan; ^4^ Department of Psychiatry Yoshino Hospital Tokyo Japan; ^5^ Department of Psychiatry Asahiyama Hospital Sapporo Japan; ^6^ Department of Psychiatry National Hospital Organization Saigata Medical Center Joetsu Japan; ^7^ Department of Health Promotion National Institute of Public Health Wako Japan

**Keywords:** alcohol dependence, nonalcoholic beer, prospective study, self‐help group, smoking cessation

## Abstract

**Background:**

Relapse after inpatient treatment for alcohol dependence is a major barrier to recovery. This study evaluated one‐year drinking outcomes and their associations with self‐help group participation, nonalcoholic beer use, smoking after discharge, and other prognostic factors among Japanese men treated for alcohol dependence.

**Methods:**

We conducted a prospective 12‐month follow‐up of 198 male patients who completed a three‐month inpatient program in 2014. Drinking status, self‐help participation, nonalcoholic beer consumption, and smoking after discharge were assessed by mailed questionnaires. Time to first drink, heavy‐drinking lapse, and sustained relapse were analyzed using Kaplan–Meier estimates and multivariate Cox proportional hazards models.

**Results:**

One‐year abstinence was higher among self‐help participants (*n* = 51) than nonparticipants (*n* = 147) (52.7% vs. 36.8%, *p* = 0.019) and higher among nonusers (*n* = 143) than users of nonalcoholic beer (*n* = 55) (51.2% vs. 15.3%, *p* < 0.0001). In multivariate models, self‐help participation reduced the risk of a drinking lapse (HR 0.51, 95% CI 0.31–0.82) and use of nonalcoholic beer increased the risk (HR 2.30, 95% CI 1.54–3.44). Smoking within one month after discharge, a family history of heavy or problem drinking, and use of psychiatric medication at discharge were also associated with higher hazards. Sensitivity analyses treating dropouts as lapses did not change the results. Heavy‐drinking‐free rates and sustained‐relapse‐free rates followed similar patterns. A dose–response pattern emerged: Having all three modifiable protective behaviors (self‐help participation, no nonalcoholic beer, and nonsmoking) offered the strongest protection against lapse and relapse [drinking lapse HR 0.18 (95% CI 0.06–0.50); heavy‐drinking lapse HR 0.13 (95% CI 0.04–0.48); sustained relapse HR 0.14 (95% CI 0.04–0.48)], two factors showed intermediate protection, and a single factor alone was not significant.

**Conclusions:**

Strengthening self‐help participation and smoking cessation support at discharge is recommended, and caution is warranted regarding nonalcoholic beer as a potential jeopardy to abstinence.

## Graphical abstract

This prospective study of 198 Japanese men found that one‐year abstinence after inpatient alcohol treatment was significantly higher among those who joined self‐help groups, avoided nonalcoholic beer, and did not resume smoking. A clear dose–response pattern showed that combining all three behaviors offered the strongest protection against relapse. These findings highlight the importance of postdischarge behavioral support and caution against nonalcoholic beer use, suggesting that targeted interventions could enhance long‐term recovery outcomes in alcohol dependence treatment.

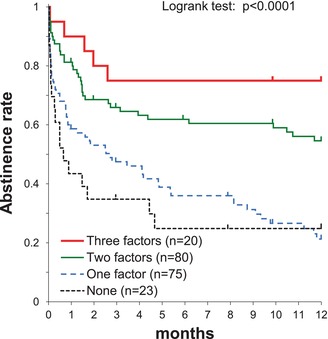

## INTRODUCTION

Alcohol dependence (AD) remains a global health challenge, with relapse after treatment discharge posing a barrier to sustained recovery. Japanese individuals are genetically and ethnically less prone to AD, with prevalence rates of 0.5% over the past 12 months and 1.0% lifetime according to an ICD‐10‐based nationwide survey (Osaki et al., 2016). Paradoxically, Japan's highly alcohol‐permissive environment, characterized by ubiquitous advertising, 24‐h retail availability, and permissive cultural norms, has led to delayed help‐seeking and entrenched social stigma for AD. Engagement with self‐help programs such as Alcoholics Anonymous (AA) or Danshukai (the Japan sobriety association) remains limited. While Japanese studies have linked participation in self‐help groups to better posttreatment outcomes including high abstinence rates (Noda et al., 2001) and improved survival (Masudomi et al., 2004; Noda et al., 1988), Japanese patients with AD often feel a strong reluctance to participate in such groups.

Nonalcoholic and low‐alcohol (up to 1.2% alcohol by volume) beverages have become increasingly popular as alternatives to traditional alcoholic drinks. However, it remains unclear how to advise heavy or problem drinkers on their use. While the use of nonalcoholic beer may help mild‐to‐moderate drinkers reduce alcohol consumption (Yoshimoto et al., 2023), emerging evidence suggests that these beverages may pose significant risks for individuals with AD who are striving to maintain sobriety (Caballeria et al., 2022).

In this prospective study, we assessed drinking status in Japanese men after completing an inpatient AD treatment program, examining its associations with two key behaviors (self‐help group participation and nonalcoholic beer use) as well as other prognostic factors (e.g., postdischarge smoking status and psychiatric medication use). By analyzing both behavioral choices and clinical background variables, we aimed to clarify how posttreatment habits influence relapse risk. These insights may inform the development of relapse prevention strategies tailored to Japanese male patients with AD.

## MATERIALS AND METHODS

This study was designed as part of a prospective drinking outcome study (Yoshimura et al., 2021) in patients admitted to Kurihama Medical and Addiction Center for AD treatment between January and December 2014. The center's standard care comprised a three‐month inpatient Alcoholism‐Rehabilitation Program (ARP). Admission to ARP required confirmation that the patient's primary treatment goal was total abstinence, ascertained at the initial consultation interview or documented in the referring physician's letter. Inclusion and exclusion criteria for this study were described elsewhere (Yokoyama et al., 2023). Briefly, 242 male patients were admitted for the first time and completed the ARP. After discharge, mailed questionnaires could not be delivered to eight patients due to unknown addresses or death within 1 month. Of the remaining 234 patients, 198 (84.6%) responded to the mailed questionnaire and were included in this analysis.

The three‐month ARP included medical management of withdrawal and physical problems, an educational curriculum, group cognitive behavioral therapy, a smoking‐cessation program, and participation in self‐help group meetings. We also hold a monthly family meeting that provides education for the families of patients.

This center admits inpatients from a wide geographic area, including distant regions. To support participation in local self‐help groups after discharge, the hospital team and patient jointly identified exact venues and times for local self‐help group meetings. After the first month of hospitalization, patients began attending weekday evening AA meetings near the addiction center or, when feasible and at the patient's request, local self‐help groups near their homes. Patients were encouraged to attend local groups during weekend home leave (Ishikawa et al., 2014).

The smoking‐cessation program, described in detail elsewhere (Yokoyama et al., 2014, 2023), was implemented under an organization‐wide smoke‐free policy and featured educational sessions. Smokers were advised to use varenicline, a smoking cessation agent, and approximately half opted for this medication during their hospital stay. At every session, research evidence highlighting the benefits of quitting smoking for long‐term alcohol abstinence and for mental and physical recovery was presented (Hurt et al., 1996; Prochaska et al., 2004; Tsoh et al., 2011; Yokoyama et al., 2014). Participants were encouraged to continue nonsmoking, attend self‐help group meetings, and receive regular outpatient care either at our hospital or at a clinic near their home after hospital discharge.

Information on age at first drinking, age at onset of habitual drinking, usual alcohol consumption, smoking status, lifestyle factors, and family history of heavy or problem drinking in grandparents, parents, or siblings was collected from participants by trained interviewers using a structured questionnaire and, when available, from their significant others. Usual alcohol consumption during the preceding year was expressed in grams of ethanol per day, calculated from the reported amounts and ethanol concentrations of consumed beverages. The presence of other major psychiatric disorders was assessed by certified clinical psychologists using the Japanese version of the Mini‐International Neuropsychiatric interview (MINI) (Otsubo et al., 2005).

Study participants were sent questionnaires monthly of the first 6 months after discharge and every 2 months from month six through Month 12. They were asked to complete and return each questionnaire. If a participant failed to respond, our technical staff called to request submission. Participant who did not return three consecutive questionnaires were classified as dropouts as of their last response. The participants were compensated with a 500 JPY (≈ 4.1 USD, 2015) prepaid card per one reply.

Participants completed a mailed questionnaire comprising eight items that assessed behaviors during the target period. Each item was framed as a binary (yes/no) question: attendance at self‐help group meetings, smoking, nonalcoholic beer consumption, any alcohol use, heavy drinking (≥ 60 g ethanol/day), outpatient visits, acamprosate use, and disulfiram use. Sample item wording was “During the target period, did you attend any self‐help group meetings? (Yes/No)” For the “any alcohol use” and “heavy drinking” items, participants were also asked to provide the date of their first drinking episode and first heavy‐drinking episode. If a date was missing, we imputed the median date of the target period. Participants reporting drinking on two consecutive assessments or failing to return the next mailed questionnaire after a drinking lapse were classified as having a sustained relapse.

All participants were diagnosed with AD by psychiatrists according to the ICD‐10 criteria (WHO, 1992). The study protocol was reviewed and approved by the Ethics Committee of the Kurihama Medical and Addiction Center (approved No. 188). All procedures were conducted in accordance with the Declaration of Helsinki, and written informed consent was obtained from each participant.

### Statistical analysis

The rates of alcohol abstinence, heavy‐drinking‐free status, and sustained‐relapse‐free status were estimated using the Kaplan–Meier method, and group differences were evaluated with the log‐rank test. Multivariate Cox proportional hazards models were used to calculate hazard ratios (HRs) with 95% confidence intervals (CIs) of selected variables on time to first drinking lapse, to first heavy‐drinking lapse, and sustained relapse. To assess the impact of study dropouts, we conducted sensitivity analyses assuming that all participants who withdrew had lapsed into drinking or heavy drinking at the time of withdrawal. We calculated the population attributable fraction (PAF) (Rockhill et al., 1998) to quantify the effect of the exploratory variables on these lapses and sustained relapse. All analyses were conducted using the SAS version 9.4 (SAS Institute, Cary, NC).

## RESULTS

Table 1 summarizes the characteristics of the 198 male patients with AD (mean age ± SD, 57.0 ± 12.8 years). Their usual daily alcohol consumption was 115.7 ± 60.8 g of ethanol. Smoking status was distributed as follows: never smokers 13.1%, current smokers 64.6%, and ex‐smokers 22.2%. A family history of heavy or problem drinking was reported by 44.9% of participants. Lifetime episodes of major psychiatric disorders were found in 43 (21.7%): 32 with affective disorder, 21 with anxiety disorder, four with antisocial personality disorder, one with psychotic disorder, and one with eating disorder. At discharge, 79 (39.9%) were prescribed medications for sobriety: acamprosate in 63 and disulfiram in 28, and 109 (55.1%) were on other psychiatric medications: sleeping drugs in 96 and antianxiety drugs in 20 (benzodiazepines in 102 [51.5%]), and antidepressants in 25, and antipsychotics in 21.

**TABLE 1:** Background characteristics in the 198 alcohol‐dependent men who had completed a three‐month inpatient alcoholism‐rehabilitation program.

Age (years)	57.0 ± 12.8
Age at first drinking	17.6 ± 3.0
≤15 years	22.2%
16–19 years	51.5%
≥20 years	26.3%
Age at the start of regular drinking	24.2 ± 7.7
≤19 years	22.2%
20–25 years	49.5%
≥26 years	28.3%
Usual alcohol intake (g ethanol/day)	115.7 ± 60.8
Most consumed alcoholic beverage	
Beer/canned chuhai (4–9%)	26.3%
Sake/wine (12–16%)	16.2%
Shochu/whiskey/other spirits (20–40%)	57.6%
Smoking status	
Never smokers	13.1%
Current smokers	64.6%
Ex‐smokers	22.2%
Living alone	29.3%
Unemployed	61.1%
Family history of heavy or problem drinking	44.9%
Lifetime episodes of other psychiatric disorders	21.7%
Medication at discharge	
Acamprosate or Disulfiram	39.9%
Other psychiatric medication	55.1%

*Note*: Data were expressed by mean ± SD or percentage values for column.

**FIGURE 1:**
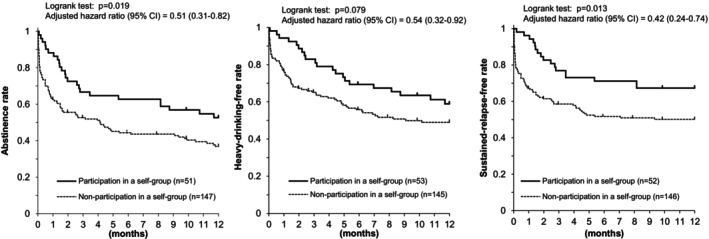
Participation in a self‐help group and drinking outcomes after a three‐month inpatient program in Japanese alcohol‐dependent men. Alcohol abstinence (left), heavy‐drinking‐free status (middle), and sustained‐relapse‐free status (right) were more frequent among subjects who participated in a self‐help group than among those who did not.

Figure 1 presents Kaplan–Meier estimates of the proportions of participants who maintained alcohol abstinence (left), those who remained free of heavy‐drinking (middle), and those who remained free of sustained drinking relapse (right) stratified by whether they participated in a self‐help group before or at the time of their first drinking lapse, heavy‐drinking lapse, sustained relapse, or through the end of follow‐up. In the abstinence, heavy‐drinking, and sustained‐relapse analyses, 51, 53, and 52 patients, respectively, participated in a self‐help group. Alcohol abstinence rates were higher among self‐help group participants than nonparticipants (52.7% vs. 36.8%, *p* = 0.019). Heavy‐drinking‐free rates (58.9% vs. 49.0%, *p* = 0.079) and sustained‐relapse–free rates (67.3% vs. 50.1%, *p* = 0.013) were also higher in participants than nonparticipants.

**FIGURE 2:**
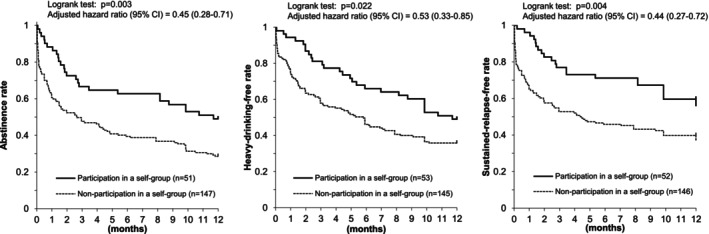
Participation in a self‐help group and drinking outcomes after a three‐month inpatient program in Japanese alcohol‐dependent men (sensitivity analysis). Sensitivity analysis assumed that all the dropout subjects drank (left), drank heavily (middle), and entered sustained relapse at the time of dropout, and that any drinking lapse at the 12‐month final response signified sustained relapse (right). The results of drinking outcomes remained essentially unchanged, supporting better drinking outcomes in those who participated in a self‐help group.

Eighteen participants were classified as dropouts in the abstinence analysis. Eight dropouts occurred in the first half of the observation period and 10 in the second half, with no clear difference by timing. Table S1 shows background characteristics for the 18 dropouts and 180 completers (113 with a drinking lapse and 67 who remained lapse‐free at one year). No factors significantly predicted dropout. Sensitivity analyses for Figure 1, which assumed all dropouts had lapsed or relapsed at the time of dropout, produced essentially unchanged results (Figure 2).

**FIGURE 3:**
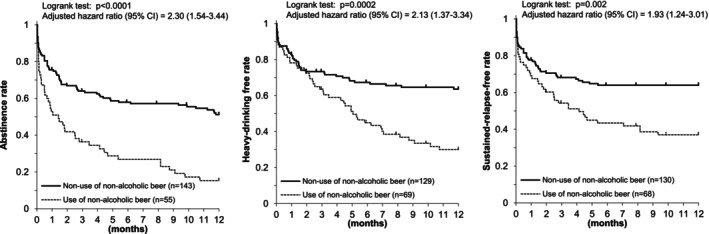
Use of nonalcoholic beer and drinking outcomes after a three‐month inpatient program in Japanese alcohol‐dependent men. Alcohol abstinence (left), heavy‐drinking‐free status (middle), and sustained‐relapse‐free status (left) were more frequent among subjects who did not use nonalcoholic beer than among those who did.

Figure 3 shows Kaplan–Meier estimates of alcohol abstinence (left), heavy‐drinking–free rates (middle), and sustained‐relapse–free rates (right) stratified by nonalcoholic beer use. Patients were classified as “users” if they consumed nonalcoholic beer before or at the time of their first drinking lapse, heavy‐drinking lapse, or sustained relapse, or if they consumed it through the end of follow‐up. In the abstinence, heavy‐drinking, and sustained‐relapse analyses, 55, 69, and 68 patients, respectively, were nonalcoholic beer users. One‐year alcohol abstinence was significantly higher among nonusers than users (51.2% vs. 15.3%, *p* < 0.0001). Heavy‐drinking–free rates (63.6% vs. 30.0%, *p* = 0.0002) and sustained‐relapse–free rates (64.0% vs. 37.0%, *p* = 0.002) were also significantly higher among nonusers than users.

**FIGURE 4:**
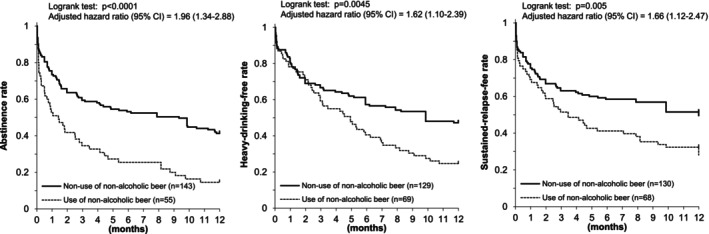
Use of nonalcoholic beer and drinking outcomes after a three‐month inpatient program in Japanese alcohol‐dependent men (sensitivity analysis). Sensitivity analysis assumed that all the dropout subjects drank (left), drank heavily (middle), and entered sustained relapse at the time of dropout, and that any drinking lapse at the 12‐month final response signified sustained relapse (right). The results of drinking outcomes remained essentially unchanged, supporting better drinking outcomes in those who did not use nonalcoholic beer.

Sensitivity analyses for Figure 3 assuming that all dropouts had lapsed or relapsed at the time of dropout yielded essentially unchanged results (Figure 4).

To examine the associations between postdischarge drinking lapse and self‐help group participation, nonalcoholic beer use, smoking status, and other background factors (Table 1), we performed a multivariate Cox proportional hazards analysis to calculate HRs with 95% CIs (Table 2). Adjusted HRs for drinking lapse were: 0.51 (0.31–0.82) for self‐help group participation, 2.30 (1.54–3.44) for nonalcoholic beer use, 1.61 (1.07–2.43) for smoking within 1 month after discharge, 1.53 (1.04–2.27) for a family history of heavy or problem drinking, and 1.53 (1.02–2.31) for psychiatric medication at discharge. No other variables were significantly associated with drinking lapse after discharge. A sensitivity analysis assuming all dropout subjects had a lapse at dropout yield essentially unchanged HRs. The PAFs for drinking lapse were −20.4% for self‐help group participation, 23.0% for nonalcoholic beer use, 20.5% for smoking within 1 month after discharge, 17.8% for a family history of heavy or problem drinking, and 20.8% for psychiatric medication at discharge.

**TABLE 2:** Drinking lapse during 12 months after a three‐month inpatient program and its determinants in Japanese alcohol‐dependent men.

	Drinking lapse				Sensitivity analysis
	Absent *n* = 85	Present *n* = 113	Multivariate HR (95% CI)	PAF	Multivariate HR^a^ (95% CI)
Age (years; per +10 years)	56.9 ± 13.1	57.2 ± 12.7	1.00 (0.83–1.19)		0.96 (0.81–1.13)
Usual alcohol intake (g ethanol/day; per +22 g/day)	104.6 ± 57.8	124.0 ± 61.9	1.06 (0.99–1.13)		1.06 (1.00–1.13)
Participation in a self‐help group before/at the time of drinking lapse or censoring	31.8%	21.2%	0.51 (0.31–0.82)	−20.4%	0.45 (0.28–0.71)
Use of nonalcoholic beer before/at the time of drinking lapse or censoring	10.6%	40.7%	2.30 (1.54–3.44)	23.0%	1.96 (1.34–2.88)
Smoking within one month after discharge	35.3%	54.0%	1.61 (1.07–2.43)	20.5%	1.69 (1.15–2.47)
Family history of heavy or problem drinking	36.5%	51.3%	1.53 (1.04–2.27)	17.8%	1.34 (0.93–1.93)
Psychiatric medication at discharge	48.2%	60.2%	1.53 (1.02–2.31)	20.8%	1.45 (1.00–2.12)

*Note*: Data were expressed by mean ± SD or percentage values for column. Multivariate HR and 95% CI by the Cox proportional hazards model. The other variables also simultaneous entered showed no significant association, that are age at first drinking, age at the start of habitual drinking, alcoholic beverage most frequently consumed, living alone, unemployed, medication of acamprosate or disulfiram at discharge, and lifetime episodes of other psychiatric disorders.

Abbreviations: CI, confidence interval; HR, hazard ratio; PAF, population attributable fraction.

^a^ Sensitivity analysis assumed that all the dropout subjects drank at the time of dropout.

A multivariate Cox proportional hazards analysis (Table 3) revealed the following HRs (95% CIs) for heavy drinking lapse: 1.08 (1.00–1.16) per 22 g/day of alcohol consumption before admission, 0.54 (0.32–0.92) for self‐help group participation, 2.13 (1.37–3.34) for nonalcoholic beer use, 1.65 (1.05–2.60) for smoking within 1 month after discharge, and 1.61 (1.02–2.55) for being prescribed psychiatric medication at discharge. No other variables were significantly associated with heavy‐drinking lapse after discharge. A sensitivity analysis assuming that all dropouts had a lapse at the time of dropout produced essentially unchanged HRs. For heavy‐drinking lapse, the PAFs were −19.7% for self‐help group participation, 26.8% for nonalcoholic beer use, 21.6% for smoking within 1 month after discharge, 12.5% for a family history of heavy or problem drinking, and 24.1% for psychiatric medication at discharge.

**TABLE 3:** Heavy drinking lapse during 12 months after a three‐month inpatient program and its determinants in Japanese alcohol‐dependent men.

	Heavy drinking lapse				Sensitivity analysis
	Absent *n* = 107	Present *n* = 91	Multivariate HR (95% CI)	PAF	Multivariate HR^a^ (95% CI)
Age (years; per +10 years)	57.4 ± 13.3	56.6 ± 12.3	0.96 (0.79–1.17)		0.90 (0.76–1.07)
Usual alcohol intake (g ethanol/day; per +22 g/day)	106.5 ± 58.5	126.4 ± 62.0	1.08 (1.00–1.16)		1.08 (1.01–1.15)
Participation in a self‐help group before/at the time of heavy drinking lapse or censoring	29.9%	23.1%	0.54 (0.32–0.92)	−19.7%	0.53 (0.33–0.85)
Use of nonalcoholic beer before/at the time of heavy drinking lapse or censoring	21.5%	50.5%	2.13 (1.37–3.34)	26.8%	1.62 (1.10–2.39)
Smoking within one month after discharge	38.3%	54.9%	1.65 (1.05–2.60)	21.6%	1.71 (1.16–2.54)
Family history of heavy or problem drinking	41.1%	49.5%	1.34 (0.87–2.08)	12.5%	1.23 (0.84–1.80)
Psychiatric medication at discharge	47.7%	63.7%	1.61 (1.02–2.55)	24.1%	1.37 (0.93–2.01)

*Note*: Data were expressed by mean ± SD or percentage values for column. Multivariate HR and 95%CI by the Cox proportional hazards model. The other variables also simultaneous entered showed no significant association, that are age at first drinking, age at the start of habitual drinking, alcoholic beverage most frequently consumed, living alone, unemployed, medication of acamprosate or disulfiram at discharge, and lifetime episodes of other psychiatric disorders.

Abbreviations: CI, confidence interval; HR, hazard ratio; PAF, population attributable fraction.

^a^ Sensitivity analysis assumed that all the dropout subjects drank heavily at the time of dropout.

A multivariate Cox proportional hazards analysis (Table 4) revealed the following HRs (95% CIs) for sustained relapse: 0.42 (0.24–0.74) for self‐help group participation, 1.93 (1.24–3.01) for nonalcoholic beer use, 2.00 (1.25–3.19) for smoking within 1 month after discharge, and 1.63 (1.05–2.53) for a family history of heavy or problem drinking. No other variables were significantly associated with sustained relapse after discharge. A sensitivity analysis assuming that all dropouts and any drinking lapse at the 12‐month final response signified sustained relapse produced essentially unchanged HRs. For sustained drinking relapse, the PAFs were −26.7% for self‐help group participation, 23.0% for nonalcoholic beer use, 29.5% for smoking within 1 month after discharge, 20.2% for a family history of heavy or problem drinking, and 20.6% for psychiatric medication at discharge.

**TABLE 4:** Sustained drinking relapse during 12 months after a three‐month inpatient program and its determinants in Japanese alcohol‐dependent men.

	Sustained drinking relapse				Sensitivity analysis
	Absent *n* = 110	Present *n* = 88	Multivariate HR (95% CI)	PAF	Multivariate HR^a^ (95% CI)
Age (years; per +10 years)	58.0 ± 12.9	55.8 ± 12.7	0.90 (0.74–1.10)		0.88 (0.74–1.05)
Usual alcohol intake (g ethanol/day; per +22 g/day)	107.6 ± 60.1	125.8 ± 60.5	1.07 (0.99–1.15)		1.08 (1.01–1.16)
Participation in a self‐help group before/at the time of sustained drinking relapse or censoring	31.8%	19.3%	0.42 (0.24–0.74)	−26.7%	0.44 (0.27–0.72)
Use of nonalcoholic beer before/at the time of sustained drinking relapse or censoring	23.6%	47.7%	1.93 (1.24–3.01)	23.0%	1.66 (1.12–2.47)
Smoking within one month after discharge	35.5%	59.1%	2.00 (1.25–3.19)	29.5%	1.89 (1.26–2.84)
Family history of heavy or problem drinking	39.1%	52.3%	1.63 (1.05–2.53)	20.2%	1.35 (0.92–1.99)
Psychiatric medication at discharge	49.1%	62.5%	1.49 (0.94–2.38)	20.6%	1.35 (0.90–2.02)

*Note*: Data were expressed by mean ± SD or percentage values for column. Multivariate HR and 95% CI by the Cox proportional hazards model. The other variables also simultaneous entered showed no significant association, that are age at first drinking, age at the start of habitual drinking, alcoholic beverage most frequently consumed, living alone, unemployed, medication of acamprosate or disulfiram at discharge, and lifetime episodes of other psychiatric disorders.

Abbreviations: CI, confidence interval; HR, hazard ratio; PAF, population attributable fraction.

^a^ Sensitivity analysis assumed that all the dropout subjects entered sustained relapse at the time of dropout, and that any drinking lapse at the 12‐month final response signified sustained relapse.

**FIGURE 5:**
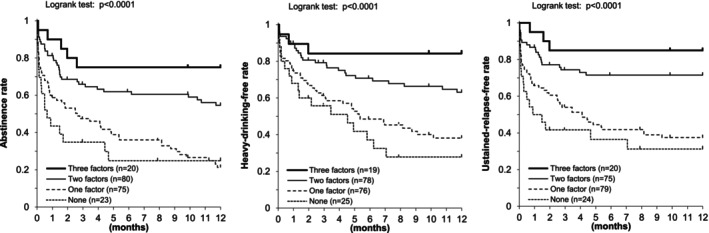
The number of modifiable protective behaviors and drinking outcomes after a three‐month inpatient program in Japanese alcohol‐dependent men. Alcohol abstinence (left), heavy‐drinking‐free status (middle), and sustained‐relapse‐free status (right) were more frequent in a dose–response relationship with increasing numbers of protective behavioral determinants present after discharge.

Simple chi‐squared tests for every pair of the three modifiable protective behaviors—self‐help attendance, avoidance of nonalcoholic beer, and nonsmoking—within each drinking outcome pattern showed no significant associations. Because the three factors were independent, we instead examined outcomes by the number of protective behaviors present; Figure 5 presents Kaplan–Meier estimates of the proportions of participants who maintained alcohol abstinence (left), those who remained free of heavy drinking (middle), and those who remained free of sustained drinking relapse (right), stratified by the number of modifiable protective behaviors present after discharge. Alcohol abstinence, heavy‐drinking‐free status, and sustained‐relapse‐free status were more frequent in a dose–response relationship with increasing numbers of protective behavioral determinants present after discharge. When the two‐factor combinations were subdivided into the three possible pairings, all three pairings produced similar Kaplan–Meier curves.

Using the group with no factors as the reference, presence of three factors conferred the strongest protective effect: drinking lapse HR 0.18 (95% CI, 0.06–0.50); heavy‐drinking lapse HR 0.13 (0.04–0.48); sustained relapse HR 0.14 (0.04–0.48). Participants with two modifiable factors also had significantly lower hazards: drinking lapse HR 0.35 (0.19–0.66); heavy‐drinking lapse HR 0.35 (0.18–0.68); sustained relapse HR 0.28 (0.14–0.57). HRs for participants with only one factor were close to unity and not statistically significant (Table 5).

**TABLE 5:** Drinking status during 12 months after a three‐month inpatient program and the number of modifiable protective behaviors in Japanese alcohol‐dependent men.

Modifiable behavioral determinants for stop drinking Self‐help group participation No use of nonalcoholic beer Nonsmoking within one month after discharge	Hazard ratio (95% CI)		
	Drinking lapse	Heavy drinking lapse	Sustained drinking relapse
Three factors	0.18 (0.06–0.50)	0.13 (0.04–0.48)	0.14 (0.04–0.48)
Two factors	0.35 (0.19–0.66)	0.35 (0.18–0.68)	0.28 (0.14–0.57)
One factor	0.91 (0.51–1.63)	0.84 (0.46–1.53)	0.86 (0.46–1.59)
None	1 (reference)	1 (reference)	1 (reference)

*Note*: Multivariate hazard ratio and 95% CI by the Cox proportional hazards model. The other variables also simultaneous entered were age at first drinking, age at the start of habitual drinking, alcoholic beverage most frequently consumed, living alone, unemployed, family history of heavy or problem drinking, medication of acamprosate or disulfiram at discharge, psychiatric medication at discharge, and lifetime episodes of other psychiatric disorders.

## DISCUSSION

This prospective study identifies key factors affecting postdischarge drinking outcomes (drinking lapse, heavy‐drinking lapse, and sustained drinking relapse) in Japanese men with AD. It highlights the protective effects of self‐help group participation, nonsmoking, and absence of psychiatric medication at discharge, and the opposing risks posed by nonalcoholic beer consumption and a family history of heavy or problem drinking. Engagement in self‐help groups significantly reduced the risks of drinking lapse and sustained relapse, underscoring their value for sustaining long‐term abstinence. In contrast, nonalcoholic beer use was strongly associated with higher risks across all drinking outcomes.

In societies where AD is prevalent within families, the benefits of recovery programs and AA participation are widely recognized, and public health initiatives vigorously support prevention. By contrast, Japan's relatively low AD prevalence has fostered permissive drinking norms, limited public awareness and understanding of the condition, and persistent stigma. Consequently, many Japanese individuals postpone seeking specialized treatment until severe mental and physical symptoms arise. Although an estimated 500,000 people in Japan met AD criteria in the past year, only around 50,000 received professional care (Osaki et al., 2016). In a matched study at our addiction center, female inpatients (median age: early forties) and age‐matched male inpatients each had an average life expectancy of only 11 years (Higuchi, 1987).

Japan offers two main self‐help options: AA, which follows a 12‐step model with spiritual elements and guarantees anonymity, and Danshukai, a weekly peer‐support group that promotes experience sharing and interpersonal support without spiritual language or anonymity, and encourages family members, primarily spouses, to attend (Higuchi & Kono, 1994). Posttreatment five‐year survival rates exceed 90% for self‐help group participants in Tokyo (Masudomi et al., 2004) and Osaka (Noda et al., 1988), compared with 60–70% for nonparticipants. Nevertheless, only one in four of our patients joined a self‐help group after discharge.

A US survey of 4014 people with AD found that 25.6% sought treatment, and about 80% of those attended 12‐step programs, where participation more than doubled the hazard rate ratio for one‐year abstinence compared with formal treatment alone (Dawson et al., 2006). Similarly, in our cohort, self‐help group attendance was associated with an approximately twofold higher HR for maintaining one‐year abstinence.

In Japan, there is currently no systematic way to link hospitals and treatment facilities with self‐help groups. Research shows that when outpatient clinics or inpatient wards call AA offices and arrange for AA members to meet patients at medical facilities, participation rates rise (Timko & DeBenedetti, 2007; Vederhus et al., 2014). One Japanese study found that arranging telephone calls between Danshukai members and patients during outpatient visits increased the odds ratio (4.4) of attending Danshukai meetings (Ino et al., 2018). At our addiction center, implementing a targeted intervention—in which the hospital team collaborated with patients to identify local self‐help group locations and schedules and actively encouraged attendance—increased inpatient participation in local groups from 25% to 44% during the program (Ishikawa et al., 2014). Community Reinforcement Approach (CRA) is a behavioral therapy that systematically arranges family, friends, work, and social activities to reduce the relative attractiveness of drinking and promote abstinence (Azrin et al., 1982; Hunt & Azrin, 1973). The abovementioned inpatient interventions to promote self‐help group participation may be considered part of CRA, and confirmation of self‐help group attendance via the present follow‐up questionnaire likely contributes to participation support. Furthermore, adapting other CRA elements—role‐play to practice in‐session skills and active outreach to family, friends, and employers—would probably increase participation rates. These findings suggest that establishing formal referral pathways within medical institutions and tailoring self‐help programs to the Japanese context are essential.

Nonalcoholic or low‐alcohol beverages are viewed favorably by drinkers as alternatives for social situations such as driving or a harm‐reduction tool, and using nonalcoholic beer may help low‐risk drinkers reduce their overall alcohol consumption (Yoshimoto et al., 2023). However, several studies suggest that these beverages may be hazardous for individuals with AD who are attempting to maintain sobriety (Caballeria et al., 2022). Such drinks can increase cravings in problem drinkers or those with AD (Kaplan et al., 1983; Long & Cohen, 1989). Alcohol‐related cues—including taste and smell—can trigger physiological responses similar to actual alcohol intake, particularly in severe cases with AD (Kaplan et al., 1985; Kareken et al., 2004; Oberlin et al., 2013). The pronounced negative impact of nonalcoholic beer consumption on drinking outcomes observed in this study suggests that alcohol‐dependent men aiming for abstinence should be warned about the potential dangers of nonalcoholic beer and advised to avoid its consumption.

Smoking within one month after discharge also emerged as an independent predictor of drinking outcomes, aligning with a previous review finding that a positive treatment outcome in AUD is facilitated by quitting smoking and countered by relapse to smoking (van Amsterdam & van den Brink, 2022). This finding suggests that integrating smoking cessation interventions into alcohol abstinence programs may improve long‐term drinking outcomes. With respect to treatment of smoking in subjects with AD, a recent meta‐analysis of RCTs showed that varenicline significantly reduced short‐term smoking (Guo et al., 2021). In our smoking cessation program, varenicline was recommended for smokers, and about half of the smokers chose to use it during the inpatient program (Yokoyama et al., 2014, 2023). Although heavier smokers were more likely to choose the use of varenicline, the nonsmoking rates of its users during hospitalization and within 1 month of discharge were significantly higher than that of nonusers. Our previous analysis of the present subjects revealed, never‐ or ex‐smokers and those who quit smoking within 1 month of discharge achieved significantly higher 12‐month alcohol abstinence (45.1–59.0% vs. 30.0%) than sustained smokers, with adjusted hazard ratios for drinking lapse of 0.57 and 0.41, respectively (Yokoyama et al., 2023). The several interpretations are possible about these findings; smoking is a trigger for alcohol consumption; alcohol consumption promotes smoking; or those who took seriously the advice that quitting smoking would help them quit drinking aimed to quit both smoking and drinking.

The risks of lapse and relapse fell progressively as patients adopted more modifiable protective behaviors (self‐help group attendance, avoiding nonalcoholic beer, and nonsmoking after discharge), suggesting additive or synergistic effects. Two behaviors produced a clear protective effect, while any single behavior alone was not statistically significant in this sample. Likely mechanisms are complementary: self‐help provides social support and coping skills, avoiding nonalcoholic beer reduces conditioned cues, and smoking cessation lowers shared neurobehavioral risks. Combining these strategies is more effective than any one alone.

Psychiatric comorbidities were prevalent in our cohort: nearly one in five patients had a lifetime history of mood or anxiety disorders. This may partly explain the high rate of psychiatric‐medication prescriptions at discharge (55.1%), which itself was associated with increased risks for drinking lapse and heavy‐drinking lapse. These results suggest that psychological distress and the need for pharmacological support identify individuals at greater risk for returning to problematic drinking behavior. This finding aligns with recent data from the same hospital, where a large‐scale study of 4116 male inpatients with AD revealed that 20% had a history of mental health issues, primarily mood disorders, and 15% had attempted suicide, underscoring the importance of early psychiatric intervention and integrated care strategies for this population (Yokoyama et al., 2025). In a self‐reported survey of 4302 Danshukai members, Akazawa et al. (2010) found that 21.6% of respondents had attempted suicide: 19.0% before joining Danshukai and only 5.3% after, suggesting that participation in self‐help groups may substantially reduce the likelihood of suicide attempts.

In the United States, addiction specialists avoid benzodiazepine agonists after alcohol withdrawal due to abuse and overdose risks when taken with alcohol (Brower, 2015), whereas long‐term use of benzodiazepines for insomnia in AD patients was common in Japan at that time, reflecting clinicians' low resistance and limited awareness of dependence hazards and high patient expectation for medication. This easy access may harm sobriety, so we now use behavioral therapies and, if necessary, orexin receptor antagonists as first‐line agents for insomnia in AD patients.

Although bidirectional causal relationships may exist between these factors and the drinking outcomes, the PAFs for nonalcoholic beer use, self‐help group participation, postdischarge smoking, and psychiatric medication (primarily benzodiazepines for sleep and antidepressants) each accounted for approximately 20% of drinking outcomes. This suggests that more intensive behavioral interventions and better management of complications could substantially improve outcomes in AD.

Previous studies have shown that a family history of AD is associated with a more recurrent course of the disorder (Farmer et al., 2022; Milne et al., 2009). The present study similarly found higher HRs for drinking lapse and sustained relapse among Japanese individuals with AD who have a family history of heavy or problem drinking. These findings suggest that genetic and environmental factors tied to familial history may influence the severity of AD (Farmer et al., 2022).

Time‐to‐event analyses may not capture the trajectory of drinking and recovery well. Indeed, 25 participants reported a first drinking lapse and then returned to abstinence in the subsequent questionnaire response. We therefore introduced “sustained drinking relapse,” defined as drinking on two consecutive assessments or failing to return the next mailed questionnaire after a drinking lapse. We categorized postabstinence drinking into three patterns—drinking lapse, heavy‐drinking lapse, and sustained drinking relapse—and performed sensitivity analyses for each. Although these three patterns represent similar events, evaluating sustained drinking relapse provides an additional perspective on treatment efficacy.

The study's strengths include the prospective assessment over 12 months and quantification of combined behavioral determinants with hazard estimates for multiple drinking outcomes. Sensitivity analyses ensured robustness of the results even under dropout assumptions. Nonetheless, several limitations must be acknowledged. First, the observational design limits the ability to establish causal relationships. Rather than being randomly assigned, participants chose to join a self‐help group, quit smoking, and refrain from drinking nonalcoholic beer. Therefore, not only does the choice itself have a positive effect on drinking prognosis, but it may also be related to the high level of motivation to quit drinking among those who made the choice. Second, the study relied on self‐reported data regarding use of alcohol beverages and nonalcoholic beer, smoking status, and participation in self‐help groups. These variables are subject to recall bias and social desirability bias, especially in a population undergoing treatment for AD. Future studies employing objective measures such as biochemical markers or validated behavioral assessments would strengthen data reliability. Third, the study has limited generalizability because it examined the outcomes after a three‐month inpatient program that is uncommon in the United States; this reflects Japan's universal insurance and a clinical pattern in which patients often enter treatment only after severe psychiatric or medical deterioration. The long inpatient stay and high baseline severity may have driven the outcomes, so it is unclear whether findings apply to extended outpatient or day‐treatment programs. Treatment selection, gender differences, and cultural factors may also affect relapse pathways and limit broader applicability. Lastly, the psychiatric background of the participants is complex. The presence of multiple comorbid psychiatric conditions and the simultaneous use of various psychiatric medications complicate attempts to isolate their individual effects on relapse outcomes.

In conclusion, the use of nonalcoholic beer and postdischarge smoking were consistently associated with increased drinking risks, whereas engagement in self‐help groups offered protective effects. These insights call for more nuanced posttreatment strategies that consider behavioral substitutes, social support structures, and psychiatric comorbidities in relapse prevention planning. Patients should avoid smoking and consuming nonalcoholic beer to support their abstinence. Participation in a self‐help group is the most evidence‐based method of recovering from AD, but more active recommendations for patients are desired.

## FUNDING INFORMATION

This research was supported by Health Labour Sciences Research Grant, Japan (H26‐seishin‐ippan‐006) and Intramural Research Grant for Neurological and Psychiatric Disorders of National Center of Neurology and Psychiatry (25–2).

## CONFLICT OF INTEREST STATEMENT

None declared.

## Supporting information


Table S1.

## Data Availability

The data that support the findings of this study are available upon request from the corresponding author. The data are not publicly available due to privacy or ethical restrictions.
