# Supplementary material for: Self‐help group participation, avoidance of nonalcoholic beer, and nonsmoking independently predict better drinking outcomes in Japanese alcohol‐dependent men
Source: Alcohol Clin Exp Res (Hoboken). 2026 Feb 1;50(2):e70227. doi: 10.1111/acer.70227 (PMC12861170; doi:10.1111/acer.70227)
Supplement: Supplementary file 1 — Table S1. [file ACER-50-0-s001.docx]

| Supplementary Table 1. Baseline characteristics by dropout status: completers (first drinking lapse or one-year lapse-free) versus dropouts (before any potential first drinking lapse) | | | |  |  |
| --- | --- | --- | --- | --- | --- |
|  | Subjects | |  |  |  |
|  | Completers | Dropouts | P |  |  |
|  | n=180 | n=18 |  |  |  |
| Age (yrs) | 57.2±12.7 | 55.4±14.1 | 0.58 |  |  |
|  |  |  |  |  |  |
| Usual alcohol intake (g ethanol/d) | 115.3±60.7 | 119.8±62.8 | 0.81 |  |  |
|  |  |  |  |  |  |
| Most consumed alcoholic beverage |  |  |  |  |  |
| beer/low-malt beer | 12.8% | 33.3% | 0.15 |  |  |
| canned chuhai | 12.2% | 5.6% |  |  |  |
| wine | 2.8% | 0.0% |  |  |  |
| sake | 13.9% | 11.1% |  |  |  |
| shochu | 46.7% | 27.8% |  |  |  |
| whiskey/other spirits | 11.7% | 22.2% |  |  |  |
|  |  |  |  |  |  |
| Age at first drinking (yrs) | 17.5±3.1 | 18.1±2.2 | 0.46 |  |  |
| Age at the start of regular drinking (yrs) | 24.0±7.4 | 27.1±10.2 | 0.11 |  |  |
|  |  |  |  |  |  |
| Cigarette smoking |  |  |  |  |  |
| Never smoking | 12.8% | 16.7% | 0.76 |  |  |
| Current smoking | 64.4% | 66.7% |  |  |  |
| Ex-smoking | 22.8% | 16.7% |  |  |  |
|  |  |  |  |  |  |
| Living alone | 29.4% | 27.8% | 1 |  |  |
| Unemployed | 61.1% | 61.1% | 1 |  |  |
|  |  |  |  |  |  |
| Family history of heavy/problem drinking | 46.7% | 27.8% | 0.14 |  |  |
|  |  |  |  |  |  |
| Lifetime episodes of other psychiatric disorders | 22.8% | 11.1% | 0.37 |  |  |
|  |  |  |  |  |  |
| Medication at discharge |  |  |  |  |  |
| Acamprosate or Disulfiram | 38.9% | 50.0% | 0.45 |  |  |
| Other psychiatric medication | 55.6% | 50.0% | 0.80 |  |  |
|  |  |  |  |  |  |
| Data were expressed by mean ± SD or percentage values for column. | | | | |  |
| P values are by Fisher's exact test for percentage values or Student t-test  or Mann-Whitney U test for continuous variables. | | | | | |
